# MiR-128 inhibits the osteogenic differentiation in osteoporosis by down-regulating SIRT6 expression

**DOI:** 10.1042/BSR20191405

**Published:** 2019-09-24

**Authors:** Jindong Zhao, Shaohui Liu, Wenhui Zhang, Linying Ni, Zhenming Hu, Zhigang Sheng, Bo Yin

**Affiliations:** 1 Department of Spinal Surgery, The Fifth Hospital of Harbin, Harbin City 150040, Heilongjiang Province, P.R. China; 2 Department of Orthopedic, The third affiliated hospital of Harbin Medical University, Harbin City 150040, Heilongjiang Province, P.R. China; 3 Department of Orthopedic, The First Affiliated Hospital of Chongqing Medical University, Chongqing City 400000, P.R. China

**Keywords:** miR-128, osteoblast differentiation, osteoporosis, SIRT6

## Abstract

**Background:** MicroRNAs (miRNAs) are involved in the regulation of osteogenic differentiation and chondrification *in vivo*. The purpose of the present study was to explore the potential mechanism of miR-128 in osteoporosis (OP).

**Methods**: Quantitative real-time PCR (qRT-PCR) was used to determine the expression of miR-128 in femoral neck trabecular bones of OP patients (*n*=40) and non-OP patients (*n*=40). C2C12 cells were transfected with miR-128 mimic or inhibitor to determine the effect of miR-128 on osteoblastic differentiation of C2C12 cells. Bioinformatics and luciferase reporter genes were used to determine the molecular mechanism of miR-128 in osteoblastic differentiation of C2C12 cells.

**Results**: The qRT-PCR results showed that the expression level of miR-128 in bone samples of OP patients was significantly higher than that of non-OP patients, while miR-128 was significantly down-regulated during the osteogenic differentiation of C2C12 cells. In addition, the results showed that overexpression of miR-128 significantly inhibited the mRNA and protein expression levels of osteocalcin (OC), alkaline phosphatase (ALP) and collagen I type-α1 (COL1A1) in C2C12 cells, while miR-128 inhibitor could reverse this effect. Bioinformatics analysis and dual-luciferase reporter assay found that silencing information regulatory protein 6 (SIRT6) was a direct target of miR-128. The qRT-PCR and Western Blot results found that miR-128 significantly down-regulated the mRNA and protein expressions of SIRT6. Furthermore, silencing SIRT6 significantly inhibited the promoting effect of the miR-128 inhibitor on the expression of osteoblast markers.

**Conclusion**: The above results confirmed that miR-128 inhibited osteoblast differentiation in OP by down-regulating SIRT6 expression, thus accelerating the development of OP.

## Introduction

Osteoporosis (OP) is a systemic metabolic bone disease, and the main pathological features were bone loss, bone microstructure change, bone brittleness increase, and fracture prone [1]. OP occurs most frequently in postmenopausal women and the middle-aged and elderly population, which is called primary OP [2]. OP is characterized by high morbidity and mortality, which can easily lead to hip fracture, seriously affect their lives and health [3]. The primary pathogenesis of OP is the disorder of bone metabolism, which results in a decrease in bone tissue due to the insufficient activity of osteoblasts and increased bone resorption [4]. Osteoblasts are important cells involved in bone remodeling, and their osteogenic activity is directly related to OP [5]. Osteoblasts are derived from the differentiation of mesenchymal stem cells (MSCs) [6], bone morphogenetic protein (BMP) 2 (BMP-2) is a cytokine that induces the differentiation of mesenchymal cells into osteoblasts by BMP family, which can not only promote the differentiation of osteoblasts *in vivo* but also act on the differentiation of osteoblasts *in vitro* [7]. C2C12 is a mouse cell line with the potential to differentiate into myoblasts, which can be induced to differentiate into osteoblasts by BMP-2 [8]. It is a cell model for the *in vitro* study of the molecular mechanism of bone formation [9].

MicroRNAs (miRNAs) are a class of endogenous non-coding, single-stranded RNA molecules with a length of 16–29 nt, which are involved not only in physiological processes such as cell growth, differentiation, metabolism, and apoptosis but also in pathological processes such as the occurrence and development of tumors [10]. Currently, a variety of miRNAs have been confirmed to be involved in maintaining bone metabolism balance; for example, miR-133a and miR-204 can inhibit osteoblast differentiation [11]. MiR-128 is an miRNA abundant in the brain and abnormally expressed in a variety of malignant tumors, such as glioma, osteosarcoma, non-small cell lung cancer (NSCLC) and gastric cancer [12,13]. Hu et al. [14] found that miR-128 could inhibit the occurrence and development of NSCLC by inhibiting the expression of vascular endothelial growth factor C (VEGF-C), thereby inhibiting lymphangiogenesis and angiogenesis. However, VEGF has been found to promote the proliferation of osteoblasts [15], which suggests that miR-128 may also be related to the proliferation and differentiation of osteoblasts. Silencing information regulatory protein 6 (SIRT6) belongs to the NAD^+^-dependent class III histone deacetylases (SIRT 1–7) family and is involved in the occurrence and development of aging, inflammation, and tumors [16,17]. However, studies on the action mechanism of miR-128 and SIRT6 in OP osteoblastic differentiation are relatively scarce. The C2C12 cell line is a typical pluripotent mesenchymal precursor cell line that possesses the potential to differentiate into myoblasts, chondroblasts, and osteoblasts [18,19]. In this study, the relationship between the expression of miR-128 in bone tissue and OP was determined by quantitative real-time PCR (qRT-PCR). Meanwhile, BMP-2-induced differentiation of C2C12 cells was used as the cell model to study the effect of miR-128 and SIRT6 on osteoblast differentiation in OP, and to explore the possible molecular mechanism.

## Materials and methods

### Materials

#### Organizational collection

Postmenopausal women with OP fractures (*n*=40) and with non-OP fractures (*n*=40) were selected, which all received hip replacement surgery. According to the T-score of BMD, the patients were divided into two groups: the normal group (T-score ≥ −1.0) and the OS group (T-score ≤ −2.5). All patients had no history of other diseases and were informed in writing. The present study was approved by The Fifth Hospital of Harbin. The cervical area of the femoral neck bone fragments was extracted from patients and then cut into small pieces. After PBS buffer washing three times, these bone fragments were kept at −80°C.

#### Reagents

The bioactive recombinant human BMP-2 (HY-P7006) was obtained from MCE. GAPDH antibody (ab181602), SIRT6 antibody (ab62739), alkaline phosphatase (ALP) antibody (ab95462), osteocalcin (OC) antibody (ab93876), collagen I type-α1 (COL1A1) antibody (ab34710), and horseradish peroxide–conjugated goat anti-rabbit IgG secondary antibody (ab6721) were obtained from Abcam. Sirt6-siRNA (sc-63028), miR-128 mimic/inhibitor and its negative control were purchased from Santa Cruz Biotechnology.

### Methods

#### C2C12 cell lines

C2C12 cell lines were purchased from the American Type Culture Collection (Manassas, VA, U.S.A.). In the present study, the C2C12 cell line was selected as the cell model of osteogenic differentiation. C2C12 cells were cultured in DMEM (Gibco) containing 10% fetal bovine serum, 100 μg/ml streptomycin, and 100 U/ml penicillin. C2C12 cells were treated with 2 nM BMP2 for 24 h to induce osteoblastic differentiation. C2C12 cells were transfected with Lipofectamine 3000, and the specific steps were followed according to the kit instructions.

#### The qRT-PCR assay

Total RNA was extracted from tissues and cells using TRIzol reagent (Invitrogen), and the specific procedures followed the kit instructions. The Primer Script™ RT kit was used to reverse transcribe the total RNA into cDNA. Primers were amplified using miScript SYBR-Green PCR kit, and the amplification conditions were 95°C for 5 min (45 circles), 95°C for 15 s, 60°C for 15 s, and 72°C for 20 s. *GAPDH* was used as the internal reference gene of mRNA, and *U6* was used as the internal reference gene of miRNA. The 2^−ΔΔ*C*^_T_ method was used to calculate the relative expression levels of genes.

#### Western blot assay

Total proteins were extracted from the cells using RIPA cell lysate and PMSF (100:1). Ten percent SDS/PAGE was used to separate the sample proteins, and then the proteins were transferred on to the NC membrane. NC membrane was closed using 5% dried skim milk solution for 30 min, and then incubated with the primary antibody at 4°C overnight. Next, the protein bands were incubated with the secondary antibody at room temperature for 1 h. The ECL chemiluminescence solution was used to color the protein bands.

#### ALP activity assay

The ALP activity in C2C12 cells was determined by the ALP staining kit (Beyotime), and the specific procedures followed the kit instructions. In brief, after fixing with 95% ethanol (v/v), the transfected C2C12 cells were incubated with the substrate solution. After incubation, cells were stained with ALP staining solution for 20 min. The absorbance value at 405 nm was determined by a microplate reader, and the ALP activity in cells was calculated according to the kit instructions.

#### Luciferase reporter gene assay

The miRanda (http://www.microrna.org), TargetScan (http://www.targetscan.org), and PicTar databases (http://pictar.mdc-berlin.de/) were used to predict the potential targets for miR-128. C2C12 cells with a density of 5 × 10^4^ cells/well were incubated in 24-well plate for 24 h. Then, 50 ng pRL-TK, 500 ng luciferase reporter gene, and 50 pmol miRNA-128 mimic or inhibitors were added to each well. After transfection for 48 h, was determined using the dual-luciferase reporter assay system (Promega Corporation, Madison, WI, U.S.A.) was used to detect the luciferase activity of cells. Firefly luciferase was used as the internal standard.

#### Statistical analysis

All data were expressed as mean ± standard deviation (SD). In the present study, SPSS 22.0 software was used for statistical analysis, and Student’s *t* test and one-way ANOVA were used for statistical significance analysis. Spearman was used for correlation analysis. *P*<0.05 indicated a significant difference.

## Results

### Determination of miR-128 expression levels in bone tissues and C2C12 cells

**Figure 1:**
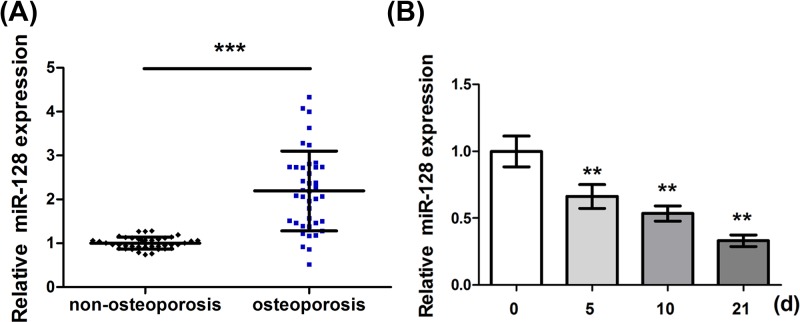
Determination of miR-128 expression levels in bone tissues and C2C12 cells (**A**) Determination of the expression of miR-128 in femoral neck trabecular bones of OP patients (*n*=40) and non-OP patients (*n*=40) by qRT-PCR. (**B**) Determination of the expression of miR-128 in C2C12 cells treated with BMP-2 at days 0, 5, 10, and 21 by qRT-PCR. **P<0.01, ***P<0.001.

In the present study, qRT-PCR was used to determine the expression of miR-128 in femoral neck trabecular bones of OP patients (*n*=40) and non-OP patients (*n*=40). The results showed that the mRNA expression of miR-128 in OP group was remarkably higher than that in the non-OP group (Figure 1A, *P*<0.001), indicating that miR-128 was highly expressed in OP bone tissues. In addition, since the C2C12 cell line can differentiate into osteoblasts, the osteoblastic differentiation cell model of C2C12 cells induced by BMP-2 was established in the present study. The expressions of miR-128 in C2C12 cells treated with BMP-2 at days 0, 5, 10, and 21 were determined by qRT-PCR. The results showed that with the prolongation of BMP-2 treatment time, the expression level of miR-128 in C2C12 cells presented a gradually declining trend (Figure 1B, *P*<0.01), which indicated that miR-128 was lowly expressed in the osteoblastic differentiation of C2C12 cells.

### Effect of miR-128 on osteoblastic differentiation of C2C12 cells

**Figure 2:**
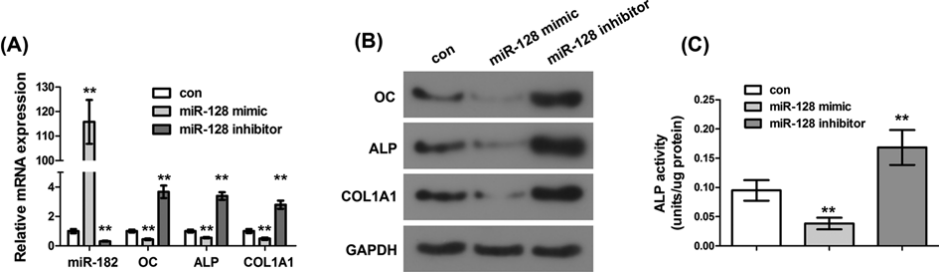
Effect of miR-128 on osteoblastic differentiation of C2C12 cells (**A**) Determination of the mRNA expressions of miR-128, OC, ALP, and COL1A1 in C2C12 cells transfected with miR-128 mimic or inhibitor by qRT-PCR. (**B**) Determination of the protein expressions of miR-128, OC, ALP, and COL1A1 in C2C12 cells transfected with miR-128 mimic or inhibitor by Western blot. (**C**) Determination of the ALP activity in C2C12 cells. **P<0.01.

OC, ALP, and COL1A1 are key markers of osteoblasts, and their expression levels directly reflect the degree of osteoblastic differentiation. To investigate the effect of miR-128 on osteoblast differentiation of C2C12 cells, we transfected C2C12 cells with miR-128 mimic or inhibitor and measured the mRNA and protein expression levels of OC, ALP, and COL1A1 in the cells. The results showed that compared with the control group, miR-128 mimic significantly increased the mRNA expression of miR-128 in C2C12 cells (Figure 2A, *P*<0.01), while miR-128 inhibitor significantly inhibited the mRNA expression of miR-128 in C2C12 cells (Figure 2A, *P*<0.01), which indicated that the cell transfection was successful. In addition, after transfection with miR-128 mimic, the mRNA and protein expressions of OC, ALP, and COL1A1 in C2C12 cells were significantly lower than those in the control group (Figure 2A,B, *P*<0.01), while miR-128 inhibitor significantly caused an opposite effect. Furthermore, the ALP activity assay showed that compared with the control group, the ALP activity in C2C12 cells transfected with miR-128 mimic was remarkably decreased (Figure 2C, *P*<0.01), while miR-128 inhibitor significantly alleviated this inhibitory effect, which was consistent with the mRNA and protein expressions of ALP. The above results suggested that miR-128 participated in and inhibited the osteoblastic differentiation of C2C12 cells.

### Identification of SIRT6 as the target gene of miR-128 in C2C12 cells

**Figure 3:**
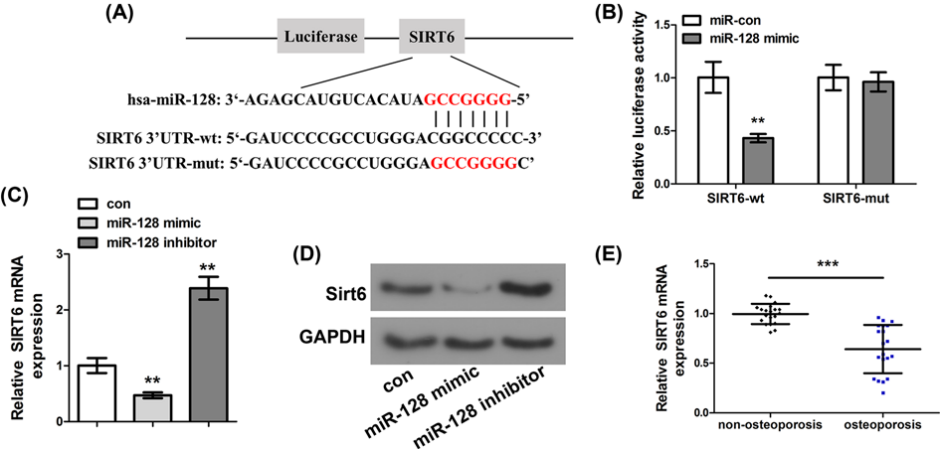
Identification of SIRT6 as the target gene of miR-128 in C2C12 cells (**A**) Schematic diagram of predicted binding sites of SIRT6 in 3′-UTR of miR-128. (**B**) Determination of the double luciferase activity of C2C12 cells transfected with SIRT6-mut or SIRT6-wt and miR-128 mimic or miR-con. (**C**) Determination of the mRNA expression level of SIRT6 mRNA in C28/I2 cells transfected with miR-128 inhibitor or mimic by qRT-PCR. (**D**) Determination of the protein expression level of SIRT6 mRNA in C28/I2 cells transfected with miR-128 inhibitor or mimic by Western blot. (**E**) Determination of the mRNA expression of SIRT6 in OP and non-OP bone tissues was determined by qRT-PCR. **P<0.01, ***P<0.001.

In this experiment, biological information was used to predict that SIRT6 was a possible target of miR-128 in C2C12 cells (Figure 3A). Meanwhile, SIRT6-wt or SIRT6-mut was co-transfected with miR-128 mimic or control into C2C12 cells to verify the binding effect between SIRT6 and miR-128. The results showed that after co-transfection with miR-128 mimic, the luciferase activity of SIRT6-wt group was significantly lower than that of SIRT6-mut group (Figure 3B, *P*<0.01). Moreover, the mRNA expression of SIRT6 in C28/I2 cells transfected with miR-128 inhibitor or mimic was determined by qRT-PCR. The results showed that inhibiting the expression of miR-128 significantly up-regulated the mRNA expression of SIRT6 in C28/I2 cells (Figure 3C, *P*<0.01). Furthermore, Western Blot showed that the protein expression of SIRT6 was also inhibited by miR-128 (Figure 3D). In addition, we also measured the mRNA expression of SIRT6 in OP and non-OP bone tissues. The results showed that the mRNA expression level of SIRT6 in OP bone tissue was significantly lower than that in non-OP bone tissue (Figure 3E, *P*<0.001), indicating that SIRT6 was poorly expressed in OP bone tissue. Pearson test was also used to investigate the relationship between SIRT6 and miR-128. A negative correlation was found the mRNA expressions between them, analyzed by qRT-PCR assay (Supplementary Figure S1). The above results indicated that SIRT6 was the miR-128 direct target gene in C2C12 cells.

### Effect of SIRT6 on osteoblastic differentiation of C2C12 cells inhibited by miR-128

**Figure 4:**
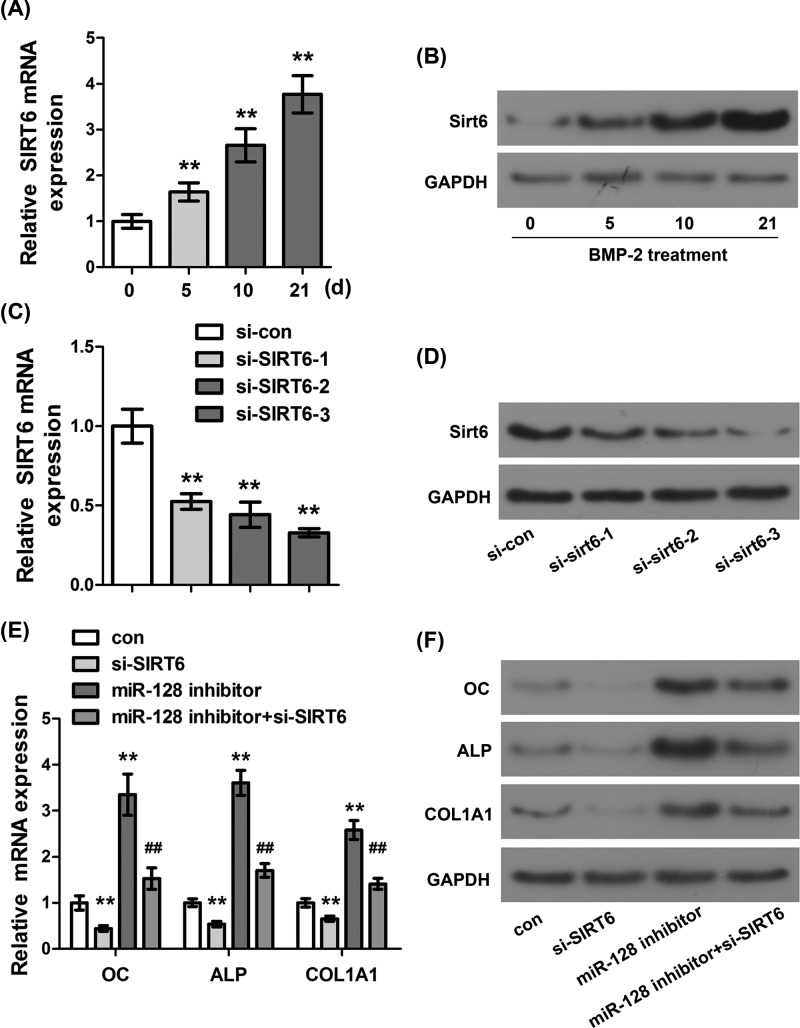
Effect of SIRT6 on osteoblastic differentiation of C2C12 cells inhibited by miR-128 (**A**) Detection of the mRNA expression level of SIRT6 in C2C12 cells induced by BMP-2 at different times by qRT-PCR. (**B**) Detection of the protein expression level of SIRT6 in C2C12 cells induced by BMP-2 at different times by Western Blot. (**C**) Determination of the mRNA expression level of SIRT6 in C2C12 cells transfected with si-SIRT6 by qRT-PCR. (**D**) Determination of the protein expression level of SIRT6 in C2C12 cells transfected with si-SIRT6 by Western Blot. (**E**) Determination of the mRNA expression levels of OC, ALP, and COL1A1 in C2C12 cells transfected with si-SIRT6 or/and miR-128 inhibitor by qRT-PCR. (**F**) Determination of the protein expression levels of OC, ALP, and COL1A1 in C2C12 cells transfected with si-SIRT6 or/and miR-128 inhibitor by Western Blot. **P<0.01, ^##^*P*<0.01 compared to miR-128 inhibitor group.

To investigate whether SIRT6 was involved in the osteogenic differentiation of C2C12 cells inhibited by miR-128, the mRNA and protein expressions of SIRT6, OC, ALP, and COL1A1 in C2C12 cells were measured. Supplementary Figure S2 provided the schematic of the functional mechanism of miR-128 on the regulation of osteoblast differentiation. MiR-128 could target SIRT6 and down-regulate the expressions of OC, ALP, and COL1A1. Through this regulation mechanism, the differentiation of osteoblast could be greatly inhibited. First, we measured the mRNA and protein expressions of SIRT6 in C2C12 cells induced by BMP-2 at days 0, 5, 10, and 21, respectively. The results showed that with the extension of BMP-2 induction time, the mRNA and protein expressions of SIRT6 in C2C12 cells presented an increasing trend (Figure 4A,B, *P*<0.01), indicating that SIRT6 was highly expressed in the process of osteoblastic differentiation of C2C12 cells. In addition, the mRNA and protein expression levels of SIRT6 in si-SIRT6 or si-con transfected C2C12 cells were determined. The results showed that the mRNA and protein expression levels of SIRT6 in the cells transfected with si-SIRT6 were significantly lower than those in the control group (Figure 4C,D, *P*<0.01), indicating that si-SIRT6 successfully inhibited the expression of SIRT6. Moreover, in order to investigate the effect of SIRT6 on the osteogenic differentiation of C2C12 cells inhibited by miR-128, we measured the mRNA and protein expressions of OC, ALP, and COL1A1 in the C2C12 cells transfected with si-SIRT6 or/and miR-128 inhibitor. The results found that, compared with the control group, silencing SIRT6 significantly inhibited the mRNA and protein expressions of OC, ALP, and COL1A1 in C2C12 cells (Figure 4E,F, *P*<0.01), while miR-128 inhibitor reversed this inhibitory effect to some extent. As mentioned above, miR-128 could inhibit the osteoblastic differentiation of C2C12 cells by down-regulating the expression of SIRT6.

## Discussion

Studies have found that the weakening of osteoblast function is one of the main pathogenesis of OP [20]. MSCs are a kind of monoclonal adult stem cells that can differentiate into multiple directions such as myoblast, lipoblast, osteoblasts, and chondroblasts [21]. C2C12 cell line is a typical multipotent mesenchymal precursor cell line that can differentiate into osteoblasts under BMP-2 induction [22]. In this study, the BMP-2-induced differentiation of C2C12 cells was used as a cell model to investigate the functional mechanism of miR-128 in OP osteoblast differentiation. The qRT-PCR results showed that miR-128 was down-regulated during the osteogenic differentiation of C2C12 cells, which suggested that miR-128 might inhibit the osteogenic differentiation of C2C12 cells.

OC, ALP, and COL1A1 are characteristic markers secreted by osteoblasts during osteoblast differentiation [23]. OC is a specific protein secreted by osteoblasts and is a sign of functional differentiation of osteoblasts [24]. COL1A1 is a marker of the beginning of the proliferation phase of osteoblasts, while ALP is a marker of early differentiation of osteoblasts, which can directly reflect the degree of differentiation of osteoblasts [25]. Zhang et al. [26] reported that overexpression of miR-221 could lead to down-regulation of crucial markers of osteoblasts, including OC, ALP, and COL1A1, thus inhibiting the differentiation of osteoblasts. Du et al. [27] found that miR-375 could down-regulate the expression of osteoblast marker molecules OC, ALP, and COL1A1 by targeting RUNX2, and ultimately inhibit the differentiation of osteoblasts while inhibiting the expression of miR-375 could enhance the expressions of OC, ALP, and COL1A1. In the present study, we transfected C2C12 cells with miR-128 mimic or inhibitor and measured the mRNA and protein expression levels of osteoblast markers OC, ALP, and COL1A1 in the cells. The results showed that compared with the control group, the mRNA and protein expressions of OC, ALP, and COL1A1 in C2C12 cells transfected with miR-128 mimic were significantly reduced, and the activity of ALP was also significantly reduced, while the miR-128 inhibitor significantly reversed this effect. Based on the above results, miR-128 might be involved in and inhibit the osteoblastic differentiation of C2C12 cells.

SIRT6 is involved in inflammatory, aging, and tumor processes in the body [28–31]. In order to investigate the functional mechanism of miR-128 in OP osteoblast differentiation, the present study used biological information and dual-luciferase gene report to speculate that SIRT6 was a direct target of miR-128 in C2C12 cells. At the same time, inhibiting the expression of miR-128 could significantly up-regulate the mRNA and protein expressions of SIRT6 in C2C12 cells. In addition, it was also found that the expression of SIRT6 was down-regulated in OP bone tissue. Therefore, SIRT6 was speculated to be a direct target of miR-128 in C2C12 cells. Furthermore, we measured the mRNA and protein expression changes of SIRT6, OC, ALP, and COL1A1 in C2C12 cells to investigate whether SIRT6 was involved in the osteogenic differentiation of C2C12 cells inhibited by miR-128. First, it was found that SIRT6 was highly expressed in the osteoblastic differentiation of C2C12 cells. Moreover, we determined the mRNA and protein expressions of OC, ALP, and COL1A1 in si-SIRT66 or/and miR-128 inhibitor transfected C2C12 cells. The results showed that silencing SIRT6 significantly inhibited the mRNA and protein expressions of OC, ALP, and COL1A1 in C2C12 cells, while miR-128 inhibitor to some extent reversed this inhibitory effect. These results suggested that miR-128 could inhibit the osteoblastic differentiation of C2C12 cells by down-regulating the expression of SIRT6. Due to the limited patients amount from our hospital, our sample size (40) is not that large. However, we will be continuously working on the examinations of miR-128’s role in OP using more patient samples in the future.

## Conclusion

In conclusion, the present study assessed the functional mechanism of miR-128 in the osteoblast differentiation of OP. The results confirmed that miR-128 inhibited osteoblast differentiation in OP by down-regulating SIRT6 expression, thus accelerating the development of OP, which provided a theoretical basis for the targeted treatment of OP.

## Availability of data and materials

The analyzed datasets generated during the study are available from the corresponding author on reasonable request.

## Supporting information

**Supplementary Figure S1:** 

**Supplementary Figure S2:** 

## Abbreviations

ALP: alkaline phosphatase
BMP: bone morphogenetic protein
COL1A1: collagen I type-α1
miRNA: microRNA
MSC: mesenchymal stem cell
NSCLC: non-small cell lung cancer
OC: osteocalcin
OP: osteoporosis
qRT-PCR: quantitative real-time PCR
SIRT6: silencing information regulatory protein 6
