# Supplementary figures and images for: MiR-128 inhibits the osteogenic differentiation in osteoporosis by down-regulating SIRT6 expression
Source: Biosci Rep. 2019 Sep 24;39(9):BSR20191405. doi: 10.1042/BSR20191405 (PMC6757182; doi:10.1042/BSR20191405)

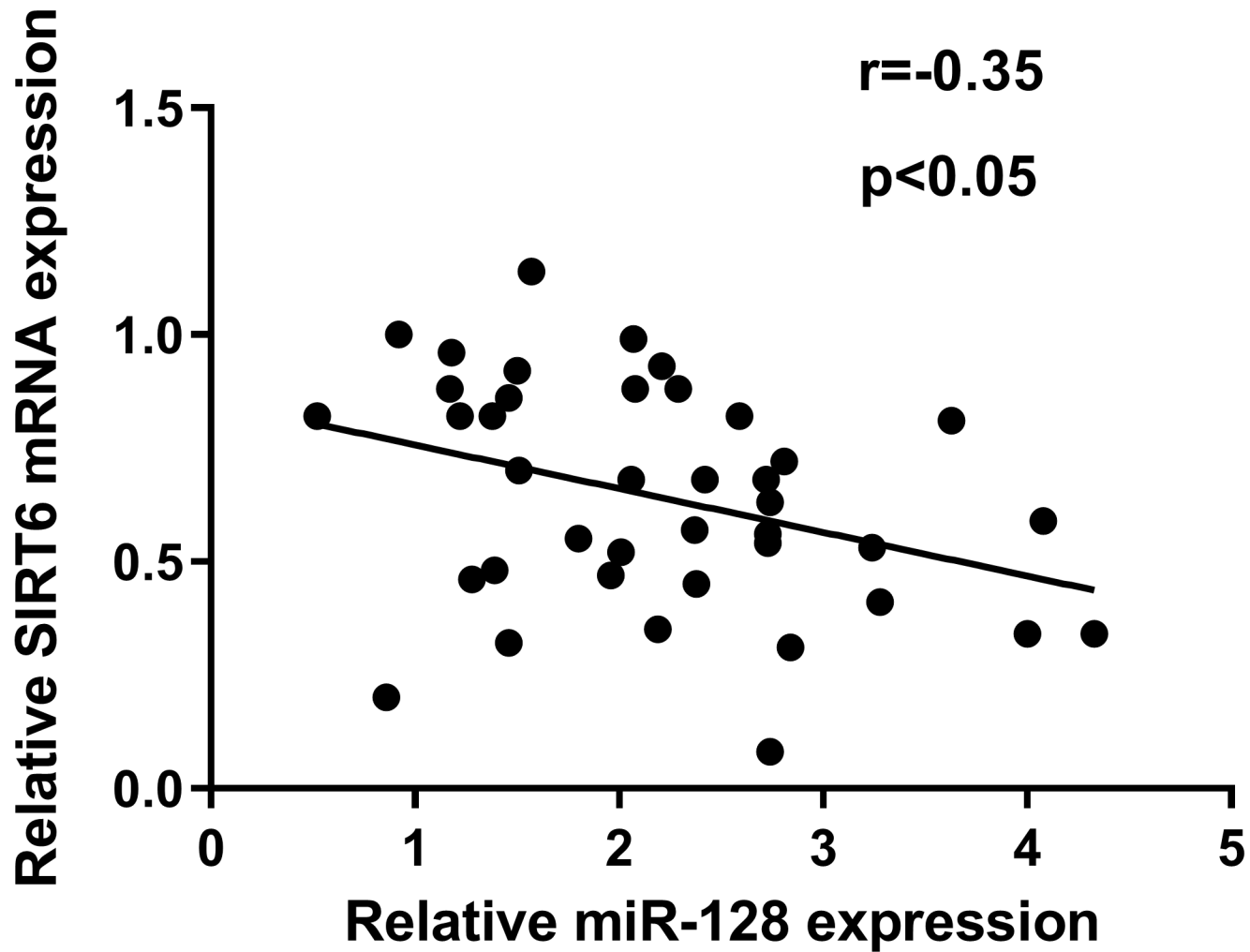

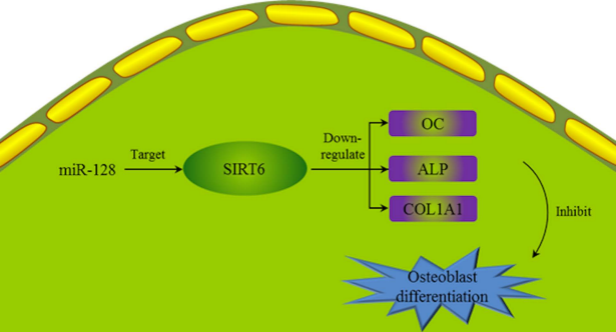

Supplement: Supplementary file 1 [file bsr20191405_Supp1.pdf]
